# Substance P and Alpha-Calcitonin Gene-Related Peptide Differentially Affect Human Osteoarthritic and Healthy Chondrocytes

**DOI:** 10.3389/fimmu.2021.722884

**Published:** 2021-08-27

**Authors:** Sabine Stöckl, Annett Eitner, Richard J. Bauer, Matthias König, Brian Johnstone, Susanne Grässel

**Affiliations:** ^1^Department of Orthopaedic Surgery, Experimental Orthopaedics, Center for Medical Biotechnology, University of Regensburg, Regensburg, Germany; ^2^Department of Trauma, Hand and Reconstructive Surgery, Experimental Trauma Surgery, Jena University Hospital, Friedrich-Schiller-University Jena, Jena, Germany; ^3^Department of Physiology, Jena University Hospital, Friedrich Schiller University Jena, Jena, Germany; ^4^Department of Oral and Maxillofacial Surgery, Center for Medical Biotechnology, University Hospital Regensburg, Regensburg, Germany; ^5^Department of Orthopedics, University Medical Center Regensburg, Asklepios Klinikum Bad Abbach, Bad Abbach, Germany; ^6^Department of Orthopaedics and Rehabilitation, Oregon Health & Science University, Portland, OR, United States

**Keywords:** osteoarthritis, SP, αCGRP, sensory nervous system, chondrocyte metabolism, ERK, cAMP, AKT

## Abstract

Osteoarthritis (OA) is a degenerative joint disease that not only causes cartilage loss but also structural damage in all joint tissues. Joints are innervated by alpha-calcitonin gene-related peptide (αCGRP) and substance P (SP)-positive sensory nerve fibers. Alteration of sensory joint innervation could be partly responsible for degenerative changes in joints that contribute to the development of OA. Therefore, our aim was to analyze and compare the molecular effects of SP and αCGRP on the metabolism of articular chondrocytes from OA patients and non-OA cartilage donors. We treated the cells with SP or αCGRP and analysed the influence of these neuropeptides on chondrocyte metabolism and modulation of signaling pathways. In chondrocytes from healthy cartilage, SP had minimal effects compared with its effects on OA chondrocytes, where it induced inflammatory mediators, inhibited chondrogenic markers and promoted apoptosis and senescence. Treatment with αCGRP also increased apoptosis and senescence and reduced chondrogenic marker expression in OA chondrocytes, but stimulated an anabolic and protective response in healthy chondrocytes. The catabolic influence of SP and αCGRP might be due to activation of ERK signaling that could be counteracted by an increased cAMP response. We suggest that a switch between the G-subunits of the corresponding receptors after binding their ligands SP or αCGRP plays a central role in mediating the observed effects of sensory neuropeptides on chondrocytes.

## Introduction

Although osteoarthritis (OA) is the world’s most common musculoskeletal disorder, research efforts have not yet been able to define its exact etiology (1, 2). Changes in peripheral joint innervation may be partly responsible for degenerative alterations in joint tissues that contribute to the development of OA and to increased pain sensation. Sensory nerve fibers, releasing substance P (SP) and alpha-calcitonin gene-related peptide (αCGRP), innervate synovium, trabecular and subchondral bone, bone marrow, periosteum and fracture callus. In addition to their classical neurological features, trophic effects that are critical for joint tissue and bone homeostasis under physiological and pathophysiological conditions have been noted (3, 4). In the context of bone remodeling during murine fracture repair, we demonstrated that absence of SP results in only a slight reduction of bone resorption rate but concomitantly in a critical reduction of bone formation and mineralization rate (5), whereas the absence of αCGRP increased the number of dysfunctional mature osteoblasts (6). Unlike bone, healthy cartilage does not contain blood vessels nor is it innervated. However, there is evidence that during OA progression, innervation occurs into cartilaginous tissues of the joints (3, 7). Suri et al. have localized SP- and αCGRP-positive nerve fibers in the articular cartilage of OA patients. They hypothesize that during the pathogenesis of OA, fine unmyelinated nerves grow into joint structures through vascular channels, mainly from subchondral bone breaching through the tidemark rather than coming from synovium or periosteum (8). Of note, vascularization and innervation of the non-calcified articular cartilage zones have been found in a wide range of histological OA stages and are not restricted to end-stage OA (3). Neither the mechanisms that drive innervation of cartilage in OA, nor its impact on the course of the disease have been determined. It is known that cartilage metabolism is modulated and influenced by neurotransmitters released either from nerve fibers located in neighboring tissue or directly from chondrocytes (3, 4, 9). Chondrocytes express SP and αCGRP as well as the receptors for both neurotransmitters, and are responsive to peripheral neuronal stimuli (3, 10). Thus, it is of great interest to understand the effects of sensory nerve fibers and their neurotransmitters on joint OA pathology and how they affect chondrocytes from degraded cartilage in comparison to chondrocytes from healthy cartilage. This study aimed to analyze the influence of the sensory neuropeptides, SP and αCGRP, on chondrocytes from OA-patients and non-OA, healthy donors, in terms of cell vitality and metabolism, chondrogenic and inflammatory processes and the activation level of pro-inflammatory signaling pathways. Our data will contribute to a better understanding of potential new application routes using these neuropeptides or their inhibitors to extend and improve the therapy spectrum for OA patients.

## Material and Methods

### Isolation and Culture of OA- and Non-OA Chondrocytes

The experiments for this study were performed with human chondrocytes from knee cartilage of OA patients who had undergone endoprothetic surgery and knee cartilage harvested from cadaver donors with healthy (termed non-OA) cartilage. We used 16 OA-patients (6 male and 10 female) and 10 non-OA donors (6 male and 4 female) in the study. The average age of the OA patients was 62 years (+/- 9 years) and for the non-OA donors was 30 years (+/- 11 years). The use of human tissue was approved by the ethics committee at the University of Regensburg (Az: 14-101-0189, email: ethikkommission@klinik.ukr.de). The cartilage was mechanically crushed and digested by collagenase type 2 (Worthington, Lakewood, USA) for 16 h at 37°C. The chondrocytes were then seeded at a density of 12,000 cells per cm² in T175 vials (Corning, Corning, USA) and cultivated with 25 ml culture medium consisting of DMEM-F12 Ham (Sigma-Aldrich, St. Louis, USA), 10% FCS (Sigma-Aldrich, St. Louis, USA) and 1% Pen/Strep (Sigma-Aldrich, St. Louis, USA) in an incubator (Thermo Fisher Scientific, Waltham, USA) at 37°C and 5% CO_2_. After 5 days, the culture medium was refreshed for the first time. The adherent chondrocytes were expanded up to a confluence of 70 - 80% by changing medium twice a week and either frozen in liquid nitrogen for storage or directly used for further experiments either in monolayer culture (2D) or in a fibrin gel culture system (3D). All experiments with OA chondrocytes were performed at passage 2 and with non-OA chondrocytes at passage 3.

Chondrocytes were stimulated in all experimental set ups with 10^-8^ M and 10^-10^ M of either SP (Sigma-Aldrich, St. Louis, USA; #S6883) or αCGRP (Bachem, Bubendorf, Switzerland; #4013281) diluted in PBS for indicated time points.

### Fibrin Gel Culture (3D)

Fibrin gels are a three-dimensional (3D) culture systems in which the cells are embedded in a fibrin matrix stabilizing the chondrogenic phenotype. Cells are supplied by diffusion of the culture medium through this matrix. OA and non-OA chondrocytes were cultivated in fibrin gels for a period of 7 days. According to Leyh et al. 2x10 (6) cells each were suspended in 10 µl fibrinogen (Sigma-Aldrich, St. Louis, USA) and 18 µl thrombin (Baxter, Deerfield, USA) and pipetted dropwise into the middle of a well of a 24-well plate (Sarstedt, Newton, USA) (11). Cultivation was performed with daily medium change of 500 µl chondrogenic medium [composition according to (12)] including the neuropeptides SP or αCGRP in a concentration of 10^-8^M and 10^-10^M diluted in PBS or PBS as a control for the neuropeptide effects. The fibrin gel cultures were used for the isolation of RNA and the supernatants were used for ELISA-based analysis.

### Protein Extraction and Western Blot Analysis

For protein analysis, chondrocytes were cultured in 6-Well plates (20.000-30.000 cells per well) for 2-3 days and treated with SP (Sigma-Aldrich, St. Louis, USA; #S6883), αCGRP (Bachem, Bubendorf, Switzerland; #4013281), GR 82334 (10^-6^ M, Tocris Bioscience, Bristol, UK; #1670), SB268262 (10µM, Tocris Bioscience, Bristol, UK; #4314), U73122 (5 µM, Tocris Bioscience, Bristol, UK; #1268) or a combination thereof. Afterwards, cells were washed with PBS, detached with trypsin–EDTA (Merck, Darmstadt, Germany) and after washing again with PBS, harvested and lysed. Total cell lysates were prepared with RIPA buffer (Invitrogen/Thermo Fisher, Waltham, MA, USA) containing proteinase inhibitor (Roche, Munich, Germany) and phosphatase inhibitor (Roche, Munich, Germany). Protein concentration was quantified with the BCA assay (Invitrogen/Thermo Fisher, Waltham, MA, USA) and lysate aliquots containing 25–50 µg of total protein (depending on the protein of interest) were boiled for 5 min with SDS-sample buffer containing β-mercapto-ethanol (Merck, Darmstadt, Germany) and subjected to a 10–15% SDS-PAGE. After electrophoretic separation, the proteins were transferred to nitrocellulose membranes (Bio-Rad, Hercules, CA, USA) and blocked with 5% dried milk (Carl Roth, Karlsruhe, Germany) and subsequently incubated with primary antibodies for 16 h at 4°C or 1 h at room temperature (**Table 1**):

**Table 1:** Antibodies used in this study.

Antibody	Company	Dilution
rabbit monoclonal anti-p44/42 MAPK (ERK1/2)	Cell Signaling Technology (CST), Danvers, MA, USA; #4695	1:2000
rabbit monoclonal anti-phospho-p44/42 MAPK (p-ERK1/2)	Cell Signaling Technology (CST), Danvers, MA, USA; #4370	1:1000
rabbit monoclonal anti-phospho-Akt (Ser473) (D9E) XP^®^	Cell Signaling Technology (CST), Danvers, MA, USA; #4060	1:2000
rabbit monoclonal anti-Akt (pan) (C67E7)	Cell Signaling Technology (CST), Danvers, MA, USA; #4691	1:1000
rabbit polyclonal anti-CRLR/CGRPR1	Invitrogen/Thermo Fisher, Waltham, MA, USA; #BS-1860R	1:500
rabbit monoclonal anti-NK1R	Abcam, Cambridge, UK; #ab183713	1:20.000
rabbit monoclonal anti-Ramp1	Abcam, Cambridge, UK; #ab156575	1:1000
rabbit polyclonal anti-beta actin	Abcam, Cambridge, UK; #ab8227	1:5000

After washing, the membranes were incubated with rabbit HRP (horseradish peroxidase)-coupled secondary antibody (1:10,000, Jackson Immuno Research, West Grove, PA, USA). Proteins were detected using ECL detection reagents (Invitrogen/Thermo Fisher, Waltham, MA, USA). Western blot signals were analyzed densitometrically using Photoshop CS3 and the ratio between the protein of interest and ß-actin or between the phosphorylated and not phosphorylated proteins was calculated.

### RNA Isolation, Reverse Transcription and Real-Time RT-PCR From Chondrocytes Cultured in 3D

All gene expression analysis were performed with RNA isolated from fibrin gels (3D). For the isolation of RNA from fibrin gels, the MasterPure Complete RNA Purification Kit (Epicentre, Madison, USA, distributed by Biozym, Hessisch Oldendorf, Germany) was used according to Leyh et al. (11, 13). The RNA concentration was determined using a NanoDrop Spectrophotometer (260/280nm) (Thermo Fisher Scientific, Waltham, USA). To generate single-stranded cDNA, RNA was reverse transcribed with an AffinityScript QPCR cDNA Synthesis Kit (Stratagene, San Diego, CA, USA) and PCR was performed with the Mx3005P QPCR System from Agilent Technologies using Brilliant II SYBER Green qPCR Mastermix (Agilent Technologies, Santa Clara, CA, USA). Gene expression in chondrocytes were analyzed relatively according to the formula 2^-ΔΔΔCT^ using the MX3005P QPCR system (Agilent Technologies, Santa Clara, USA) and were analyzed with the MxPro QPCR software for Mx3000P and Mx3005P QPCR systems (Stratagene, La Jolla, USA). The results were calibrated to the gene expression in untreated control cells, and normalized to TBP and 18s. Primer sequences are shown in **Table 2**.

**Table 2:** qPCR primers.

Gene	Forward	Reverse
18S ribosomal RNA	CTGGATACCGCAGCTAGGAA	GAATTTCACCTCTAGCGGCG
ACAN (Aggrecan)	CTATACCCCAGTGGGCACAT	GGCACTTCAGTTGCAGAAGG
BMP4 (Bone morphogenetic protein 4)	AAGCGTAGCCCTAAGCATCA	TGGTTGAGTTGAGGTGGTCA
COL2A1 (Collagen type II alpha 1)	CCAGATGACCTTCCTACGCC	TTCAGGGCAGTGTACGTGAAC
COL9A1 (Collagen type IX alpha 1)	GAG CAC CGA CAG ATC AGC AC	AGT GGC ACC TGA GTC TGG A
COX2 (Cyclooxygenase-2)	TGC TTG TCT GGA ACA ACT GC	TGA GCA TCT ACG GTT TGC TG
ICAM-1 (Intercellular Adhesion Molecule 1)	AGCTTCTCCTGCTCTGCAAC	GACAATCCCTCTCGTCCAGT
IL1ß (Interleukin 1ß)	TAA GCC CAC TCT ACA GCT GG	GAG AGG TGC TGA TGT ACC AG
IL6 (Interleukin 6)	CAA TGA GGA GAC TTG CCT GG	GCA CAG CTC TGG CTT GTT CC
ITGA11 ((Integrin subunit alpha 11)	GCAGCAGCCTGAGCCACTAC	AGCACGACGCAAGTCTTCCTC
P16 (Cyclin-dependent kinase inhibitor 2A, CDKN2A)	CCA ACG CAC CGA ATA GTT ACG	GCG CTG CCC ATC ATC ATG
SIRT1 (Sirtuin-1)	GGA GCA GAT TAG TAGGCG GC	CCT CAG CGC CAT GGA AAA TG
SOX9 (SRY-Box Transcription Factor 9)	GTACCCGCACTTGCACAAC	TCTCGCTCTCGTTCAGAAGTC
TBP (TATA-Box Binding Protein)	GAACATCATGGATCAGAACAACA	ATAGGGATTCCGGGAGTCAT
TNF alpha (tumor necrosis factor alpha)	CAC ATT CCT GAA TCC CAG GT	TCC TTC AGA CAC CCT CAA CC
VEGFR (vascular endothelial growth factor)	TGAAAACTTTGGAAGACAGAACC	ACAGACTCCCTGCTTTTGCT

### CellTiter-Blue Cell Viability Assay

The CellTiter-Blue Cell Viability Assay (Promega, Germany) was performed to assay viability of chondrocytes stimulated with SP or αCGRP for 1, 3 or 7 days in 2D cell culture and for 7 days in 3D cell culture (fibrin gels). 10.000 cells/well were seeded into 96-well plates for 1 day of SP or αCGRP stimulation, 8.000 cells/well were seeded into 96-well plates for 3 days of SP or αCGRP stimulation and 7.000 cells/well were seeded into 48-well plates for 7 days of SP or αCGRP stimulation. Chondrocytes were daily treated with SP- or αCGRP-containing medium. Fibrin gels were prepared with 500.000 chondrocytes embedded in 2,5 µl Fibrinogen and 4,5 µl Thrombin, and treated for 7 days with SP or αCGRP with daily medium changes and addition of neuropeptides. After indicated stimulation time, the amount of resazurin reduced to resorufin (pink and highly fluorescent) was determined at 579/584 nm with a Tecan ELISA reader (Maennedorf, Switzerland).

### Proliferation – Doubling time

For assaying the cell growth, equal numbers of cells (20,000-30,000) were seeded in 6-well plates in triplicates and treated with SP or αCGRP each day. The cells of one well were detached each day and counted with a cell counter (Cedex, Roche, Germany) for up to 6 days. The doubling time was calculated using the following formula:

Doubling Time=(duration × log(2))/(log(Final Concentration)-log(Initial Concentration))

### Caspase-3/7 Assay

For assaying apoptosis, equal numbers of cells (5000) were seeded in black 96-well plates in triplicates and treated with SP or αCGRP for 1 day. Caspase-3/7 enzymatic activity was measured as an indicator of apoptosis using the Apo-ONE Homogeneous Caspase-3/7 assay (Promega, Fitchburg, WI, USA) according to the manufacturer’s instructions. A non-fluorescent caspase substrate (Z-DEVD-R110), added to the HTB94 cells, was thereby cleaved into fluorescent molecules with an emission maximum at 521 nm and measured in a microplate reader (Tecan, Männedorf, Switzerland).

### Senescence-Associated (SA)-ß-Galactosidase Assay

For assaying senescence, equal numbers of cells (5000) were seeded in 96-well plates in triplicates and treated with SP or αCGRP for 1 day. For the determination of cellular senescence, we used the Mammalian SA-β-Galactosidase Assay Kit (Thermo Fisher Scientific, Waltham, USA) according to manufacturer’s instruction. Thereby, we measured the senescence-associated β-galactosidase (SA-β-gal) activity by using a fluorometric substrate, producing a bright yellow color with a peak absorbance at 420 nm that can be quantified using a microplate reader (Tecan, Männedorf, Switzerland).

### Adhesion Assay

For measuring the adhesion of chondrocytes on cell culture dishes, 1000 cells were seeded (per well of a 96-well plate) together with SP or αCGRP. Culture medium and non-adhered cells were removed 30 min later. Cells were washed with PBS and fixed with 1% Glutaraldehyd (Merck, Darmstadt, Germany) for 30 min at rt. Crystal violet solution (in 0,02% aqua dest.) (Carl Roth, Karlsruhe, Germany) was added to the wells at rt for 15 min. After removing of the staining solution, wells were washed with deionized H_2_O and crystal violet was extracted from the cells by adding 70% ethanol for 3 h. Plates were shaken gently at room temperature for 15 min and 100 µl of the extracted crystal violet were transferred to a flat-bottom 96-well plates. Absorbance was measured at 590 nm in a microplate reader (Tecan, Männedorf, Switzerland).

### IL6 and IL1ß-ELISAs

The amount of IL6 or IL1ß in the cell culture supernatant of the fibrin gel cultures was measured according to manufacturer’s instruction using a human IL-6 ELISA (Ray Biotech, Norcross, GA, US) or a human IL-1ß ELISA (R&D, Minneapolis, MN, US). Chondrocytes in fibrin gels were daily treated with SP or αCGRP for 7 days and supernatant samples were harvested on the last day. Standards and samples are pipetted into the wells of an antibody pre-coated plate and IL-6 or IL1ß present in a sample or standard is bound by the immobilized antibody. The standard curve is used to determine the concentration of the samples. Standards and samples are measured at 450 nm in a microplate reader (Tecan, Männedorf, Switzerland).

### Collagen Type II ELISA and DMMB Assay

Chondrocytes were cultured in fibrin gels and treated daily with SP or αCGRP for 7 days. Fibrin gels were homogenized and digested with pepsin (10 mg/ml in 0.05 M acetic acid containing 0.4 M NaCl, Sigma-Aldrich, St. Louis, MO, USA) for 48 h at 4°C and further digested with elastase (1 mg/ml in Tris-buffered saline (TBS) pH 8; Serva Electrophoresis, Heidelberg, Germany) for 24 h at 4°C. Samples were stored at -20°C until they were analyzed for GAG (glycosaminoglycan) or collagen type II content by ELISA.

Collagen type II content of digested fibrin gels were measured with specific sandwich ELISA, which recognize the native conformation of collagen type II chains (Chondrex, Redmond, WA, USA), according to the manufacturer’s instructions and measured in an ELISA-reader (Tecan, Männedorf, Switzerland) at 490 nm. All measurements were performed in triplets.

GAG content of digested fibrin gels were measured by DMMB (dimethyl methylene blue) (Merck, Darmstadt, Germany) assay. DMMB specifically stains chondroitin sulphate. Aliquots (25 µl) of the sample or of a standard dilution series (1:2) of known concentrations of chondroitin sulphate A (Merck, Darmstadt, Germany), used for calibration, were mixed with 250 μl DMMB staining solution (2ml formic acid and 2g sodium formate were dissolved in distilled water, pH 3.0 adjusted with HCl; 18 mg DMMB solved in 5 mL ethanol and added) and measured in an ELISA-reader (Tecan, Männedorf, Switzerland) at 595 nm. All measurements were performed in triplicate.

### Gelatin Zymography

Gelatin zymography was performed to analyze MMP-2 or -9 activity with chondrocytes cultured either in 2D monolayer or 3D fibrin gels. For monolayer culture 50.000 cells/well were seeded into 6-well plates. Chondrocytes were cultured for 3 days with daily addition of fresh medium containing SP or αCGRP. For the last 24 hours, cells were cultured in serum-free medium containing SP or αCGRP. Fibrin gels were prepared with 500.000 chondrocytes embedded in 2,5 µl Fibrinogen and 4,5 µl Thrombin, and treated for 7 days with SP or αCGRP with daily medium changes and addition of neuropeptides Equal amount of supernatants were mixed with 5x loading buffer (20% glycerol, 2% SDS, 2mM EDTA, 0.02% bromophenol blue, 20 mM Tris/HCl, pH 8.0) and subjected to electrophoresis (10% SDS-polyacrylamide gels supplemented with 1% gelatin). Subsequently, gels were washed twice for 30 min in Triton-X 100 (2,5%) (Sigma-Aldrich, US) and in distilled water, and were developed for 1 day at 37°C in Tris/HCl (50mM), pH 8.5, containing CaCl2 (5mM). Finally, the gels were stained with Coomassie Brilliant Blue R250 (Serva, Germany) to detect protease activity. Signals were analyzed densitometrically using Photoshop CS3.

### MMP13 Western Blot

50.000 cells/well were seeded into 6-well plates. Chondrocytes were cultured for 3 days with daily addition of fresh medium containing SP or αCGRP. For the last 24 hours, cells were cultured in serum-free medium containing SP or αCGRP with or without addition of IL1ß (1ng/µl) for 24h (Thermo Fisher Scientific, Waltham, USA) known as an inducer of proteases. Fibrin gels were prepared with 500.000 chondrocytes embedded in 2,5 µl Fibrinogen and 4,5 µl Thrombin, and treated for 7 days with SP or αCGRP and with or without addition of IL1ß (1ng/µl) for 24h with daily medium changes and addition of neuropeptides. Equal amount of supernatants were mixed with 5x loading buffer (20% glycerol, 2% SDS, 2mM EDTA, 0.02% bromophenol blue, 20 mM Tris/HCl, pH 8.0) and subjected to electrophoresis. MM13 was detected using anti-rabbit polyclonal antibody to MMP13 (1:6000, 1 hour RT in 5% dry milk, Abcam, #ab39012) and rabbit horseradish peroxidise-coupled secondary antibody; 1:10.000, Jackson Immuno Research, US). Protein bands were detected with ECL detection reagents (Thermo Fisher Scientific, US).

### cAMP ELISA

For assaying the intracellular cyclic AMP (cAMP) level equal numbers of cells (20,000-30,000) were seeded in 6-well plates in triplicate. After preincubation with Rolipram (Sigma-Aldrich, St. Louis, USA) and following treatment with forskolin (Sigma-Aldrich, St. Louis, USA), SP or αCGRP, cAMP was measured using the cAMP ELISA Kit (Colorimetric) (Cell Biolabs Inc., San Diego, CA, USA) according to the manufacturer’s instruction. The cAMP-ELISA is a competitive enzyme immunoassay, using an anti-Rabbit IgG polyclonal coating antibody being adsorbed onto a microtiter plate. Cyclic AMP present in the sample or standard competes with Peroxidase cAMP Tracer for plate binding, in the presence of Rabbit Anti-cAMP Polyclonal Antibody. Following incubation and wash steps, a Peroxidase cAMP Tracer bound to the plate is detected with addition of substrate solution. The colored product formed is inversely proportional to the amount of cAMP present in the sample. The reaction is terminated by addition of a Stop solution and absorbance is measured at 450 nm. A standard curve is prepared from cAMP standard and sample concentration is then determined.

### Statistics

Statistical analysis was performed using Prism 6 (GraphPad Software Inc., San Diego, CA, USA). Results are presented in boxplots (median, min to max) or columns (mean, SD). Each assay was performed in replicates and repeated at least in three independent experiments. One sample t-tests were used to determine if the mean of the different groups (experimental group *vs.* control) were significantly different. Exact P-values were calculated. A value of p ≤ 0.05 was considered statistically significant. For the semi-quantitative analysis of Western blot intensity (or if specifically denoted), unpaired t-tests were used.

## Results

### Production of SP and αCGRP and Their Receptors NK1-R and CLRL/Ramp1 in OA- and Healthy Chondrocytes

**Figure 1:**
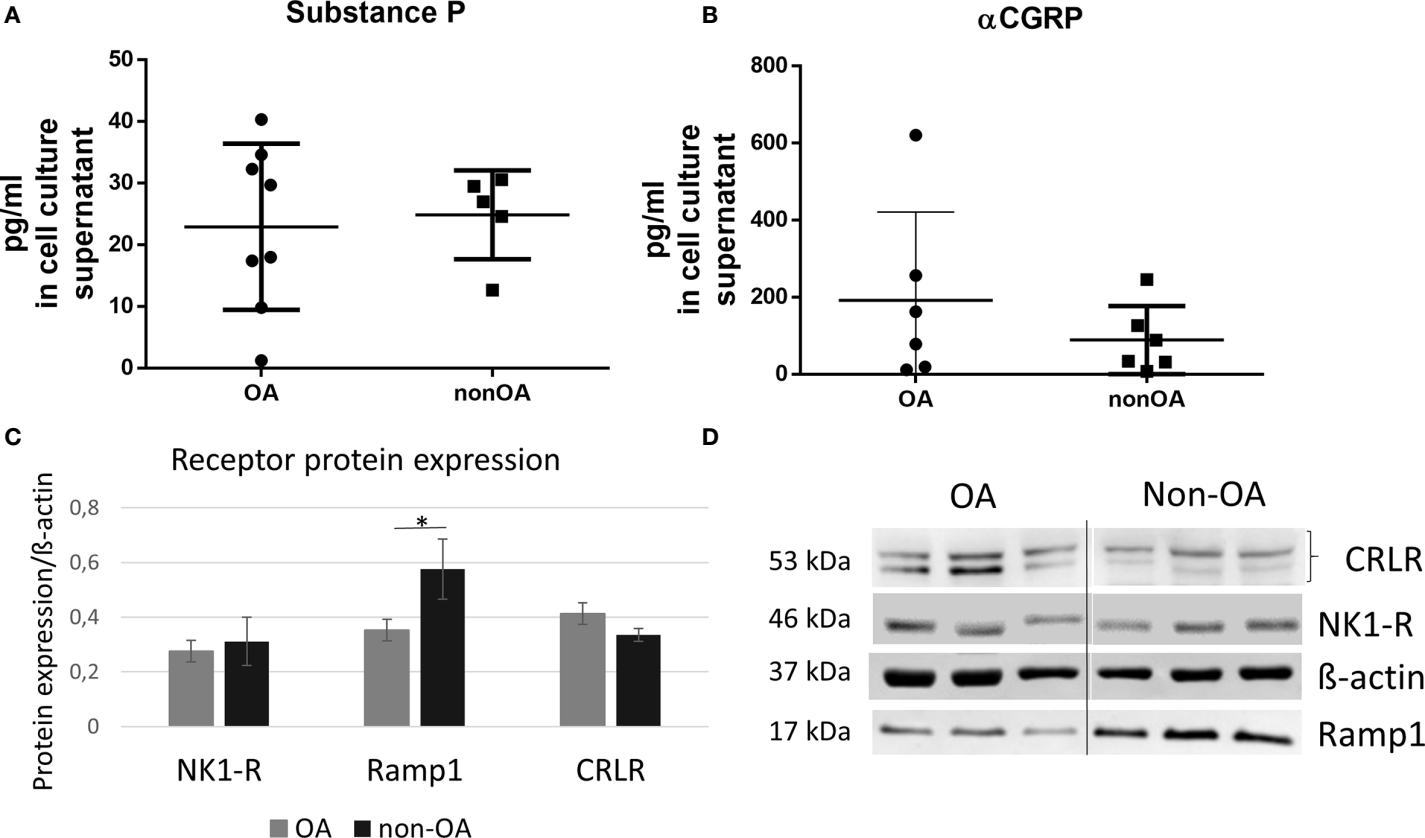
Protein biosynthesis of SP, αCGRP, NK1-R and CGRP-R (CRLR and Ramp1) in OA and non-OA chondrocytes. **(A, B)** Protein concentration of SP and αCGRP was determined in the cell culture supernatants with ELISA, n = 5-8. **(C)** Relative receptor protein expression was determined densitometrically. Expression of β-actin served as endogenous loading control, n = 3. **(D)** Representative Western Blot images showing CRLR (~53 kDa), NK1-R (~46 kDa), Ramp1 (~17 kDa) and β-actin (~37 kDa) protein bands in chondrocyte lysates from OA- and non-OA donors (n = 3 different donors for each group). Results are means +/- SD; one sample t-test *p ≤ 0,05; OA, osteoarthritis; SP, substance P; αCGRP, alpha-calcitonin gene-related peptide; NK1-R, neurokinin 1 receptor; CRLR, calcitonin receptor-like receptor; Ramp1, receptor activity modifying protein 1.

We analyzed the concentration of SP and αCGRP in the culture supernatants of chondrocytes from OA patients (OA-CH) and healthy donors (non-OA-CH). Whereas for SP no differences were found between both groups (OA-CH: 22,91 ± 4,762 pg/ml *vs.* non-OA-CH: 24,85 ± 3,218 pg/ml) (**Figure 1A**), the level of αCGRP was increased by trend in the supernatant of OA-CH (191,3 ± 93,77 pg/ml) compared to non-OA-CH (88,79 ± 36,00 pg/ml), although not statistically significantly (**Figure 1B**). The protein expression of neurokinin-1 receptor (NK1R), the receptor with the highest affinity for SP, was similar for OA- and non-OA-CH (**Figures 1C, D**). The receptor for αCGRP (CGRP-R) consists of two transmembrane protein subunits, the calcitonin receptor-like receptor (CRLR) unit and the receptor activity-modifying protein 1 (RAMP1) unit, with the latter subunit providing the specificity for αCGRP. The signal of the band corresponding to RAMP1 was significantly weaker in OA-CH, whereas the band signal intensity of CRLR in OA-CH was similar to that in non-OA-CH (**Figures 1C, D**).

OA and non-OA chondrocytes produced and secreted SP and αCGRP in comparably low concentrations (pg/ml). Non-OA chondrocytes showed higher expression of the αCGRP receptor subunit RAMP-1 compared to OA chondrocytes.

### Effects of SP and αCGRP on Viability, Proliferation, Apoptosis, Senescence and Adhesion

**Figure 2:**
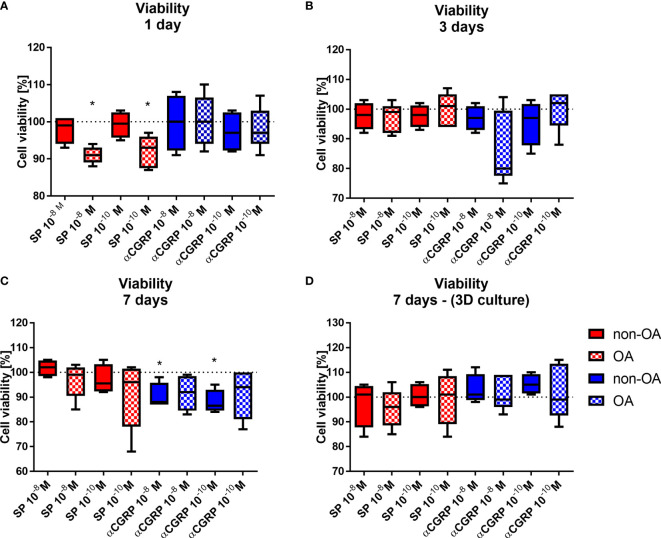
Effects of SP and αCGRP stimulation on viability of OA and non-OA chondrocytes. Chondrocytes were treated with either 10^-8^ or 10^-10^M SP or αCGRP and then compared with the respective untreated control cells (untreated controls set to 100% = dotted line). Viability was determined in monolayer culture after 1 day **(A)**, 3 days **(B)** and 7 days **(C)**. **(D)** Viability was determined in 3D fibrin gel culture after 7 days. Results show median (min to max); one sample t-test; *p ≤ 0,05; n = 5.

First, we analyzed effects of SP and αCGRP treatment on viability of OA- and non-OA chondrocytes at different culture time points. Both concentrations of SP reduced viability of OA-CH at the first day of culture but not at later culture time points compared to untreated controls. Also, both concentrations of αCGRP reduced viability of non-OA-CH at day 7 - the end point- of culture compared to untreated controls (**Figures 2A–C**). Viability of chondrocytes kept in 3D fibrin gel culture was not affected at any time point (**Figure 2D**).

**Figure 3:**
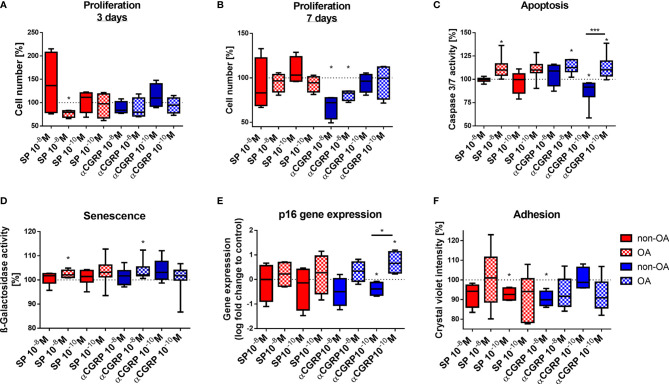
Effects of SP and αCGRP stimulation on proliferation, apoptosis, senescence and adhesion of OA and non-OA chondrocytes. Chondrocytes were treated with either 10^-8^ or 10^-10^ M SP or αCGRP and then compared with the respective untreated control cells (untreated controls set to 100% = dotted line). **(A, B)** Proliferation was determined after 3 days and 7 days of SP/αCGRP stimulation by cell counting. **(C)** Apoptosis was measured with a caspase 3/7 assay after 1 day of SP/αCGRP stimulation. **(D, E)** Analysis of SA-ß-galactosidase activity and p16 gene expression revealed the senescence status of the cells after 1 day of SP/αCGRP stimulation. **(F)** The adhesion capacity was analysed *via* crystal violet staining after 30 minutes of SP and αCGRP stimulation. Results show median (min to max); one sample t-test; *p ≤ 0,05; ***p ≤ 0,001; n = 4-7.

We compared the proliferation rate of OA- and non-OA-CH to their respective untreated control cells by counting the cell numbers after 3 and 7 days of stimulation with SP or αCGRP in two different concentrations (10^-8^M or 10^-10^M). After 3 days of stimulation with 10^-8^M SP, OA-CH had proliferated less compared with control cells (**Figure 3A**). This effect was lost after 7 days, but by then, stimulation with 10^-8^M αCGRP leads to a significantly reduced proliferation rate in OA- and non-OA-CH (**Figure 3B**).

Apoptosis was also influenced by the two neuropeptides. Stimulation with SP (10^-8^M), and also with αCGRP (10^-8^M and 10^-10^M) for 1 day, increased caspase 3/7 activity in OA-CH, indicating enhanced apoptosis in those cells. In contrast, 10^-10^M αCGRP has beneficial effects on non-OA-CH by decreasing apoptosis significantly (**Figure 3C**).

As apoptosis is often closely related to cellular senescence, we measured the senescence-associated (SA)-ß-galactosidase activity as an indicator for senescence-related processes in OA- and non-OA-CH after 1 day of addition of SP and αCGRP. OA-CH respond with an enhanced SA-ß-galactosidase activity to addition of 10^-8^M SP and αCGRP (**Figure 3D**). Even though there is no universal marker for cell senescence, most senescent cells increase expression of p16 (p16^ink4A^), a cell cycle inhibitor that targets cyclin-dependent kinases (CDKs). Thus, we analyzed the gene expression of p16 after 7 days of incubation with both neuropeptides. αCGRP exerted opposite effects on OA- and non-OA-CH. Whereas in non-OA-CH, αCGRP reduced the expression of p16, in OA-CH αCGRP induced gene expression of this senescence marker (**Figure 3E**) in comparison with untreated control cells.

Chondrocyte adhesion is critical in cell based regenerative strategies for treatment of focal cartilage defects. As SP and αCGRP might be of interest for therapeutic approaches, we investigated the adhesion behavior of OA- and non-OA-CH during stimulation with the two neuropeptides. Both neuropeptides decreased the number of adherent non-OA-CH at 30 minutes after seeding; SP in a low concentration (10^-10^M) and αCGRP in a high concentration (10^-8^M) (**Figure 3F**). As we have observed direct effects of SP and αCGRP on different cellular metabolic properties like viability, proliferation, apoptosis, senescence and adhesion in OA- and non-OA-CH, we analyzed the metabolic activity of SP- or αCGRP-treated cells using a real-time metabolic bioanalyzer (Seahorse XF). No significant differences between any groups were detected (**Supplementary Figure S1**).

Both neuropeptides affected metabolism of OA- and non-OA chondrocytes differently. SP stimulation mostly affected OA-chondrocytes whereas in non-OA chondrocytes, SP only modulated adhesion capability. Contrary, both OA- and non-OA chondrocytes responded to αCGRP stimulation.

### Expression of Chondrogenic and Mesenchymal Markers in OA- and Healthy Chondrocytes After SP and αCGRP Stimulation

**Figure 4:**
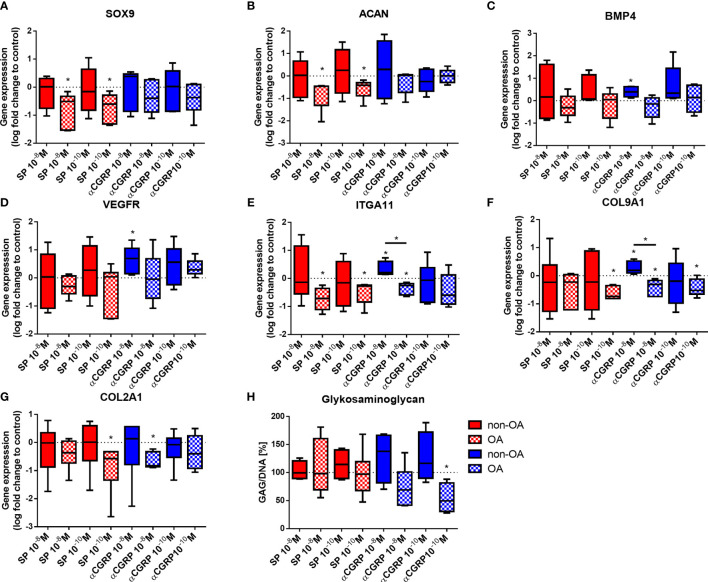
Expression of chondrogenic and mesenchymal markers in 3D fibrin gels cultured OA- and non-OA chondrocytes after stimulation with SP and αCGRP. Chondrocytes were treated with either 10^-8^ or 10^-10^ M SP or αCGRP for 7 days and then compared to the respective untreated control cells (untreated controls set to 0 or 100% = dotted line). **(A–G)** Quantitative RT-PCR analysis revealed the gene expression of SOX9, ACAN, BMP4, VEGFR, ITGA11, COL9A1 and COL2A1. **(H)** The glycosaminoglycan (GAG) content in OA and non-OA chondrocytes was analysed with a DMMB assay. Results show median (min to max); one sample t-test; *p ≤ 0,05; n = 5-6.

To detect the influence of SP and αCGRP on chondrogenic/mesenchymal marker genes, we analyzed the expression levels of SOX9, ACAN, BMP4, VEGFR, ITGA11, COL9A1 and COL2A1 (**Figures 4A–G**). The OA- and non-OA-CH were encapsulated in fibrin gels for 7 days with daily SP or αCGRP addition and the gene expression levels were compared with the respective untreated control chondrocytes. We measured decreased gene expression of SOX9, ACAN (**Figures 4A, B**) and ITGA11 (**Figure 4E**) in OA-CH after addition of 10^-8^ and 10^-10^M SP, and of COL9A1 and COL2A1 in the presence of 10^-10^M SP (**Figures 4F, G**). In contrast, the gene expression levels of BMP-4 (**Figure 4C**), VEGFR (**Figure 4D**), ITGA11 (**Figure 4E**) and COL9A1 (**Figure 4F**) were increased in non-OA-CH after 10^-8^M αCGRP addition. In contrast to the effects on non-OA-CH, the gene expression of COL2A1 (**Figure 4G**), COL9A1 (**Figure 4F**) and ITGA11 (**Figure 4E**) gene expression were lower in OA-CH after 10^-8^M αCGRP addition. COL9A1 gene expression in OA-CH was also decreased by 10^-10^M αCGRP stimulation (**Figure 4F**). The biosynthesis of collagen type II was not affected (**Supplementary Figure S2**) in either chondrocyte group after SP or αCGRP addition, but a decreased glycosaminoglycan (GAG) content in OA-CH containing fibrin gels after incubation with 10^-10^M αCGRP was observed (**Figure 4H**).

Stimulation with SP affected only gene expression in OA-chondrocytes (reduction of anabolic gene expression of SOX9, ACAN, ITGA11, COL9A1, COL2A1) but not in non-OA chondrocytes. Stimulation with αGRRP induced gene expression (BMP4, VEGFR, ITGA11, COL9A1) in non-OA chondrocytes but reduced gene expression (ITGA11, COL9A1, COL2A1) in OA-chondrocytes. However, protein synthesis of collagen II remained unaffected as did mostly GAG synthesis except in OA chondrocytes after αCGRP stimulation.

### Expression of Inflammatory Markers in OA- and Healthy Chondrocytes After SP and αCGRP Stimulation

**Figure 5:**
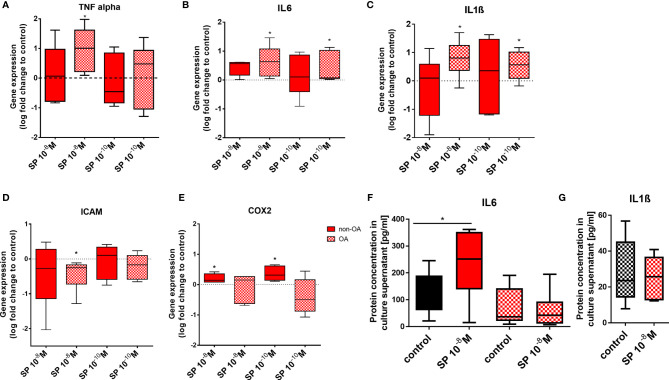
Pro-inflammatory markers in 3D fibrin gels cultured OA and non-OA chondrocytes after stimulation with SP. Chondrocytes were treated with either 10^-8^ or 10^-10^ M SP for 7 days and then compared to the respective untreated control cells (untreated controls set to 0 or 100% = dotted line). **(A–E)** Quantitative RT-PCR analysis demonstrated the gene expression of TNFα, IL6, IL1ß, ICAM and COX2. **(F, G)** Protein concentration of IL6 and IL1ß in the culture supernatants was assayed *via* ELISA. Results show median (min to max); one sample t-test; *p ≤ 0,05; n = 5-6.

SP and αCGRP effects are often related to inflammatory processes in various tissues and can influence the expression of pro-inflammatory cytokines. We therefore analyzed gene expression of TNFα, IL6, IL1ß, ICAM and COX2 (**Figures 5A–E**) and protein concentration of IL6 and IL1ß in culture supernatants (**Figures 5F, G**) after stimulation with SP or αCGRP. In OA-CH, 10^-8^M SP resulted in an increased gene expression of TNFα, IL6 and IL1ß (**Figures 5A–C**). The gene expression of IL1ß was also increased when OA-CH were subjected to a lower dose of SP (10^-10^M) (**Figure 5C**). The gene expression of the transmembrane protein ICAM-1 (CD54), a cell surface glycoprotein known for regulating leukocyte recruitment from circulation to sites of inflammation, was reduced in OA-CH after treatment with 10^-8^M SP (**Figure 5D**). In non-OA-CH, 10^-8^M and 10^-10^M SP increased the gene expression of COX2 (**Figure 5E**), a key mediator of pro-inflammatory pathways, and 10^-8^M SP increased the gene expression level of IL6 in non-OA-CH compared to control cells (**Figure 5B**). We also measured protein levels in cells with altered IL6 or IL1ß gene expression. IL6 protein amount was increased in the supernatant of non-OA-CH that had been exposed to 10^-8^M SP, in comparison with controls (**Figure 5F**). However, in the OA-CH group, neither the IL6 (**Figure 5F**) nor IL1ß (**Figure 5G**) concentrations in the supernatants were affected by 10^-8^M SP treatment. Notably, αCGRP did not affect any of the analyzed inflammatory markers neither in OA- nor in non-OA-CH (**Supplementary Figures S3A–E**). Notably, the IL1ß concentration in non-OA-CH supernatants was below the ELISA detection limit. In addition, the expression and activity of different MMPs was analyzed in OA- CH after SP stimulation. MMP-9 was not detectable at all and MMP-13 was mostly only detectable after IL1ß stimulation in 3D culture only and not affected by SP stimulation. Activity of pro- and active forms of MMP-2 was not affected by SP stimulation neither in 2D- nor in 3D culture (**Supplementary Figures S4A–E**).

Expression of pro-inflammatory genes remained unaffected by αCGRP stimulation but was induced by SP stimulation (except ICAM gene expression which was reduced) in both chondrocyte groups. In addition, SP stimulation increased IL6 concentration in culture supernatant of non-OA CH.

### Activation of cAMP-Dependent Pathways in OA- and Healthy Chondrocytes

**Figure 6:**
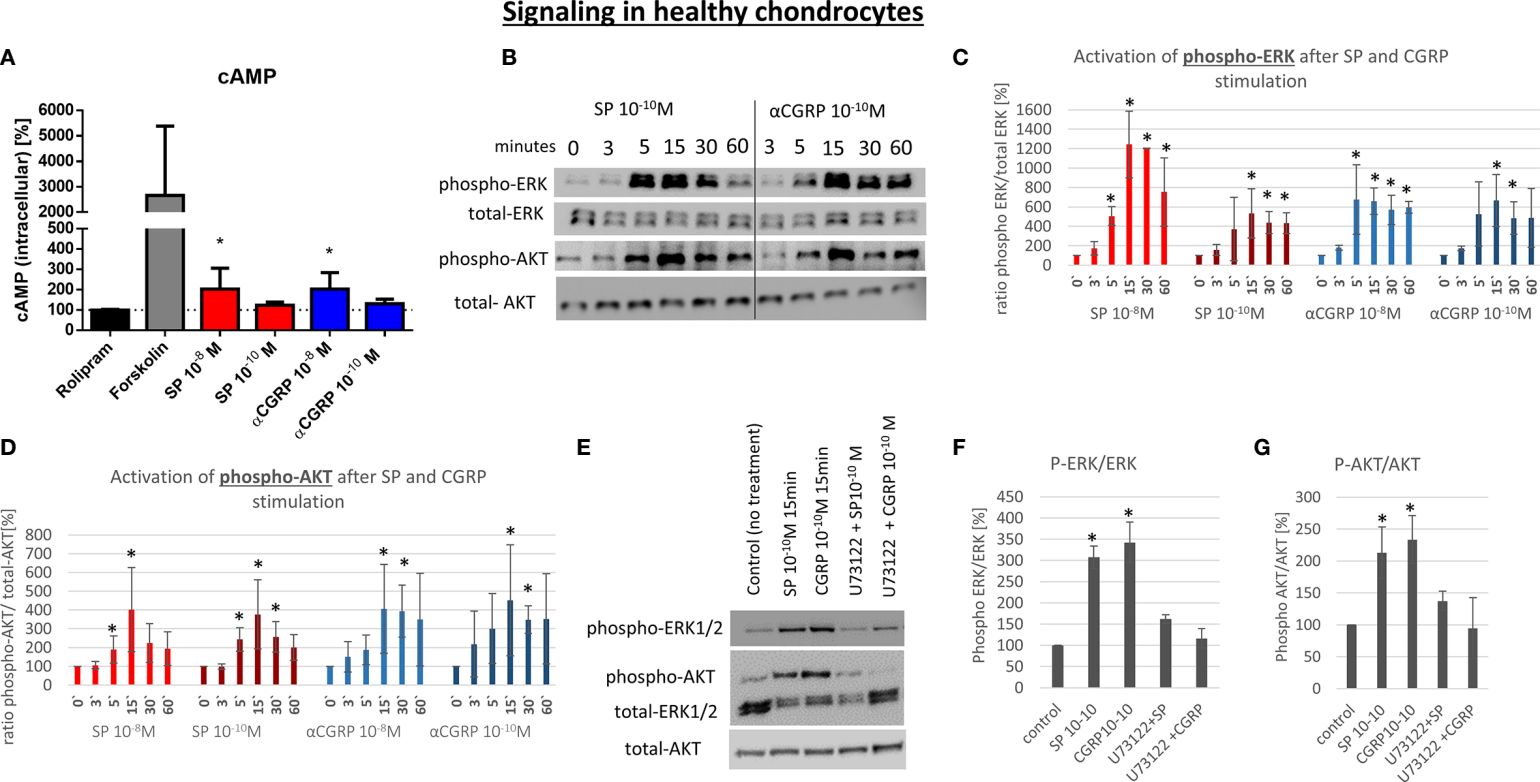
Effect of SP and αCGRP on signaling in non-OA chondrocytes. Chondrocytes were treated with either 10^-8^ or 10^-10^ M SP or αCGRP for 3, 5, 15, 30 and 60 minutes and then compared to the respective untreated control cells. **(A)** Level of cAMP was detected *via* ELISA. Cells were pre-incubated with Rolipram (phosphodiesterase 4 inhibitor) to avoid cAMP degradation, and then treated with SP, αCGRP or Forskolin (positive control for cAMP induction). The graph shows the relative amount of cAMP in relation to Rolipram-incubated cells (dotted line = 100%) in non-OA chondrocytes. n = 3. **(B)** Representative Western Blot images indicating phospho-ERK1/2 (~ 44/42 kDa), ERK1/2 (~44/42 kDa), phospho-AKT (~60 kDa) and AKT (~60 kDa) protein signals after 0, 3, 5, 15, 30 and 60 minutes of neuropeptide stimulation in non-OA chondrocytes. **(C, D)** Ratio of phospho-ERK1/2 to total-ERK1/2 and phospho-AKT to total-AKT protein concentration was determined densitometrically. Expression w/o stimulation (0’) served as control set to 100%; n = 3. **(E)** Representative Western Blot images of U73122 (= phospholipase C inhibitor) treated non-OA chondrocytes to verify G alpha q mediated AKT- and ERK1/2-signaling. **(F, G)** Ratio of phospho-ERK1/2 to total-ERK1/2 and phospho-AKT to total-AKT protein concentration was determined densitometrically. Expression w/o stimulation served as control set to 100%; n = 3; Results are means +/- SD; one sample t-test *p < 0.05.

**Figure 7:**
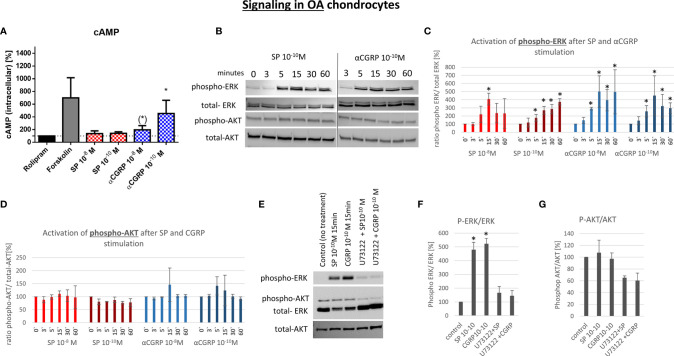
Effect of SP and αCGRP on signaling in OA chondrocytes. Chondrocytes were treated with either 10^-8^ or 10^-10^ M SP or αCGRP for 3, 5, 15, 30 and 60 minutes, and then compared to the respective untreated control cells. **(A)** Level of cAMP was detected *via* ELISA. Cells were pre-incubated with Rolipram (phosphodiesterase 4 inhibitor) to avoid cAMP degradation, and then treated with SP, αCGRP or Forskolin (positve control for cAMP induction). The graph shows the relative amount of cAMP in relation to Rolipram-incubated cells (dotted line=100%) in OA chondrocytes. n = 3. **(B)** Representative Western Blot images for phospho-ERK1/2 (~ 44/42 kDa), ERK1/2 (~44/42 kDa), phospho-AKT (~60 kDa) and AKT (~60 kDa) protein concentration after 0, 3, 5, 15, 30 and 60 minutes of stimulation in OA chondrocytes. **(C, D)** Ratio of phospho-ERK1/2 to total-ERK1/2 and phospho-AKT to total-AKT protein expression was determined densitometrically. Expression w/o stimulation (0’) served as 100% control, n = 3. **(E)** Representative Western Blot images of U73122 (= phospholipase C inhibitor) treated OA chondrocytes to verify G alpha q mediated AKT- and ERK1/2-signaling. **(F, G)** Ratio of phospho-ERK1/2 to total-ERK1/2 and phospho-AKT to total-AKT protein expression was determined densitometrically. Expression w/o stimulation served as 100% control, n = 3. Results are means +/- SD; one sample t-test, (*) p = 0,065; *p < 0.05.

G-protein-coupled receptors (GPCR) like NK1-R and CLRL/Ramp1 (CGRP-R) are able to activate different signaling pathways *via* different alpha-subunits. To elucidate the signaling mechanism in OA- and non-OA-CH after SP and αCGRP treatment, we examined the level of the second messenger cyclic adenosine-mono-phosphate (cAMP), which is known to be activated after assembling of GPCRs with Gαs subunits. To do this, we pre-incubated the cells with Rolipram, a selective inhibitor of type 4 cyclic nucleotide phosphodiesterases (PDE4), which mediates cAMP degradation. In non-OA-CH a high concentration (10^-8^M) of SP and αCGRP provoked a comparable increase in the cAMP-level (**Figure 6A**). In contrast, in OA-CH, SP did not induce alterations in the cAMP level, but αCGRP increased the amount of cAMP significantly when applied in a concentration of 10^-10^M, and by trend also at 10^-8^M (**Figure 7A**). It is noteworthy that in non-OA-CH a markedly stronger cAMP response was generated by forskolin treatment, which represents the internal positive treatment control (non-OA-CH: cAMP mean after forskolin: 2658 pM (+/-1574); OA-CH: cAMP mean after forskolin: 699 pM (+/-183)).

αCGRP stimulation provoked increased cAMP concentrations in non-OA and OA, whereas SP only induced cAMP level in non-OA-CH.

### PLC-Dependent Activation of ERK and AKT Pathways in OA- and Healthy Chondrocytes

The activation of the ERK and AKT signaling pathways is a critical step in the regulation of chondrocyte gene expression in a chronic inflammatory state. Here, we analyzed if SP or αCGRP can induce the phosphorylation of ERK or AKT in OA- and non-OA-CH. In general, non-OA-CH developed a stronger response to the treatment with SP and αCGRP than OA-CH (**Figures 6** and **7**). Both neuropeptides induced phosphorylation of ERK and AKT within 5-15 minutes without alteration of the unphosphorylated ERK and AKT protein concentration in non-OA-CH (**Figures 6B–D**).

We hypothesized that the receptors for SP and αCGRP (NK1-R and CGRP-R) can assemble not only with Gαs but also with the αq subunit (Gq) in chondrocytes, as this is often the case when ERK and AKT pathways are activated. Alpha q proteins couple to G proteins of GPCR to activate beta-type phospholipase C (PLC-β) enzymes which modulate the formation of phosphoinositol -3-phosphate (PIP3) which in turn is responsible for anchoring AKT/PKB at the membrane and thus modulates signaling. We therefore used a specific PLC-ß inhibitor (U73122) in combination with SP and αCGRP to test this hypothesis. The phosphorylation of ERK and AKT can be blocked when non-OA-CH are treated with SP and αCGRP together with U73122 (**Figures 6E–G**), indicating an involvement of PLC-ß in this signaling pathway. In OA-CH, the induction of ERK phosphorylation *via* SP and αCGRP treatment was detected within 5 minutes (**Figure 7B**), but the ratio of phospho-ERK to total ERK was lower compared with that in non-OA-CH in the presence of SP (**Figure 7C**). The phosphorylation of ERK *via* αCGRP in OA-CH was comparable with that seen in non-OA-CH (**Figure 7C**). In contrast, the AKT pathway could not be activated *via* SP or αCGRP treatment in OA-CH (**Figures 7B, D**). Treatment of OA-CH with the specific PLC-ß inhibitor U73122 in combination with SP and αCGRP lead to an inhibition of ERK phosphorylation (**Figures 7E, F**), similar to the effect seen in non-OA-CH. The unchanged levels of phospho-AKT in control, SP- and αCGRP-treated OA-CH decreased slightly after PLC-ß inhibition (**Figures 7E, G**).

To further show that the induction of ERK and AKT phosphorylation is SP- and αCGRP-specific, we used NK1-R antagonist (GR 82,334) and CGRP-R antagonist (SB 273779) for treatment of a human chondrocyte cell line (C28/I2), which reacts comparably to non-OA-CH to SP and αCGRP treatment. In both cases, the receptor antagonists were able to reduce the phosphorylation of ERK and AKT, when applied together with SP or αCGRP (**Supplementary Figures S5A, B**).

Both neuropeptides induced phosphorylation of ERK1/2 in OA- and in non-OA CH whereas AKT-phosphorylation was only induced by both neuropeptides in non-OA-CH.

## Discussion

Neurotransmitters of the sensory nervous system can influence the physiology of various cell types of the musculoskeletal system, mainly acting as trophic factors in this context (14–16). We have previously reported profound effects of SP and αCGRP on osteoblasts, macrophages and osteoclasts **(** 5, 6 **).** Treatment of bone marrow derived macrophages from tachykinin-1 knockout mice (no SP) with SP reduced proliferation and caspase 3/7 activity, while affecting osteoblast proliferation and caspase activity in a time-dependent manner (5). Mature adult αCGRP-deficient mice showed an increase of dysfunctional mature osteoblasts (6). Studies analyzing effects of the sensory neurotransmitters SP and αCGRP on chondrocyte metabolism are rare. Hence, we focused in our study on catabolic/anabolic cellular effects of the neuropeptides and compared their influence on chondrocytes from OA patients and non-OA, healthy donors. This may help to better assess the mode of SP and αCGRP effects in OA knee joints, where articular cartilage mostly consists of both osteoarthritic and healthy appearing regions (17).

We detected no difference in the concentration/expression of SP or NK1-R between OA- and non-OA chondrocytes. Taken together with the results of Im et al., who observed a significant increase of SP in the synovial fluid of OA patients (18), it can be assumed that chondrocytes are not responsible for the increased SP level in synovial fluid during OA progression. Rather, other cell types, such as synovial fibroblasts, are considered to be a major source of SP production and release (19). For αCGRP, it has been shown that the expression (20, 21) and release (22, 23) is increased in afferents from rat and murine joints affected by OA. The amount of αCGRP released from human OA chondrocytes was increased by trend in our study in comparison to chondrocytes from healthy cartilage. At the same time, protein expression of RAMP-1, the αCGRP specific subunit component of the αCGRP receptor, was significantly decreased in OA chondrocytes in comparison with non-OA chondrocytes, indicating that there may be an increased sensitivity of chondrocytes specifically to αCGRP during OA pathology.

In the present study, we observed that both, SP and αCGRP negatively affect the viability of chondrocytes, however at specific time points only, with SP decreasing viability of OA-chondrocytes and αCGRP decreasing viability of healthy chondrocytes which is reflected in increased apoptosis after SP stimulation and decreased proliferation after αCGRP treatment. These data emphasize the critical effects deriving from the environment of the cells. We demonstrated dose- and time-dependent negative effects of αCGRP on OA chondrocytes in proliferation, apoptosis and senescence, whereas in non-OA chondrocytes, the influence of αCGRP was mainly protective, with an anti-apoptotic and anti-senescence effect (reduced p16 gene expression). This is in line with previous results from our group for murine osteoblasts and osteoclasts (6). αCGRP applied in a high dose for 7 days had anti-proliferative effects on OA- and non-OA chondrocytes in our experiments, whereas a lower dose did not influence the growth rate in any of the groups. In contrast, a high SP dose decreased the proliferation of OA chondrocytes after a short period of treatment (3 days). This contrasts with the work of Opolka et al. who reported a dose-dependent increase in the proliferation of primary costal chondrocytes from newborn mice with SP (10), indicating that the effects of SP and αCGRP depend on the type and physiological status of the target cells. αCGRP effectively enhanced primary rat osteoblast proliferation (24), but exerted anti-proliferative effects in aortic and pulmonary artery smooth muscle cells (25). SP treatment stimulates proliferation in bone marrow stromal cells (15) and in fibroblasts during tendon repair (26, 27). Growth inhibiting effects of SP, as we have observed for OA chondrocytes, have also been reported, for example in hair follicle cells (28). Clinical therapeutic approaches that use autologous chondrocyte implantation for focal cartilage lesion repair demonstrated that the adhesion of chondrocytes is an essential step for successful regeneration. Based on our results, and considering those of others, we speculate that altered SP or αCGRP levels in synovial fluid or joint tissues could delay or impair engraftment of healthy chondrocytes subsequent to implantation. We observed reduced gene expression of integrin α11 in OA-chondrocytes after neuropeptide treatment whereas gene expression in chondrocytes from healthy donors was not affected. The difference between OA- and non-OA chondrocytes in terms of adhesion after SP or αCGRP treatment may thus either result from reduced expression of integrins or a switch to expression of different subsets of integrins in chondrocytes as degeneration progresses (29).

The phenotype of chondrocytes can be defined by analyzing specific markers. The variability of the expression of these markers is age and health status related, but can also be influenced by growth factors or SP or αCGRP. For SP, it is reported that it can accelerate terminal differentiation of chondrocytes (30) and it seems to be essential for cartilage homeostasis because it participates in transduction of mechanical cues through the NKR-1 (3, 31, 32). In contrast, knowledge of the effects of αCGRP on chondrocytes and cartilage biology is scarce. We demonstrated a stimulating and positive impact of αCGRP on chondrogenic marker gene expression (COL9A1, ITGA11, BMP-4) in healthy chondrocytes, whereas in OA chondrocytes, the same αCGRP concentration inhibited gene expression of these markers (COL2A1, COL9A1, ITGA11). We observed a strong inhibition of COL2A1 and COL9A1 gene expression when OA chondrocytes were treated with a low SP concentration and downregulation of SOX9, ACAN, ITGA11 after treatment with both SP concentrations. These observations are in line with recent studies showing that SP plays an important role in the process of OA cartilage degeneration (33, 34) and may imply that an increased SP level in the joints can accelerate destructive processes during OA pathology or hamper healing processes. We have previously reported that in a murine destabilization-induced osteoarthritis model the cartilage degradation was delayed in SP-deficient mice (35), underlining our observations of the catabolic influence of SP on OA chondrocytes in the present study.

In addition, expression of inflammation-related genes was increased in OA chondrocytes treated with SP, whereas non-OA chondrocytes showed little response. Pro-inflammatory cytokines, commonly secreted by immune cells, are the main driver of the low-grade inflammation during OA pathology (36). However, expression of the pro-inflammatory cytokines IL1β and TNFα, classically secreted in early OA (37), was significantly increased in OA chondrocytes after SP treatment, indicating a critical role of SP in the inflammatory response of chondrocytes. Also for other musculoskeletal tissues, such as tendons, it has been shown that SP can promote inflammation (38). We demonstrated a dose-dependent effect of SP on inflammation markers indicating the possibility that while a low SP dose does not critically alter the inflammation process in the joint, higher doses of SP have destructive effects on joint tissues by reinforcing the inflammatory milieu. It is notable that αCGRP did not affect the expression of any tested inflammation marker in both chondrocyte groups, indicating no direct influence of αCGRP on inflammatory processes *via* chondrocytes in the joints of OA patients. Nevertheless, it is likely that the peripheral actions of αCGRP in the joints do contribute to inflammation and also to joint afferent sensitization (39), as αCGRP is also linked to inflammatory pain by attenuating responses in αCGRP knock-out mice in a number of pain models (40, 41). The increase of the αCGRP concentration in OA joints, as reported by Tankano et al., does not drive specifically chondrocytes to intensify the inflammatory process but would, through other cell types such as synovial fibroblasts, trigger catabolic processes in OA joints (42).

The receptors for SP and αCGRP are classical G protein-coupled receptors (GPCR). These receptors can activate at least three types of G-proteins: Gs, which activates adenylyl cyclase (AC)/protein kinase A (PKA), the Gq/G11 family, which activates phospholipase C (PLC)/protein kinase C (PKC), and Gi, which can inhibit adenylyl cyclase (43). Activation of Gαs and cAMP exert anti-inflammatory effects, whereas activation of Gαq and PLC affect the receptor-stimulated mitogen-activated-protein-kinase (MAPK) pathway resulting in pro-inflammatory stimuli (44, 45). In our study, OA- and non-OA chondrocytes increased the production of the second messenger cAMP after αCGRP binding to its receptor, confirming the data of Edoff et al., who reported similar results when rat chondrocytes were treated with αCGRP (46). αCGRP is able to increase cAMP in a concentration-dependent manner in several cell types, as was demonstrated in neonatal dorsal root ganglion (DRG) neurons (47). Moreover, treatment with a high dose of SP increased the cAMP level in non-OA chondrocytes similar to treatment with a high dose of αCGRP. In contrast, SP did not alter the cAMP response in OA chondrocytes, indicating no anti-inflammatory signaling effects for those cells *via* SP. Interestingly, in OA chondrocytes, a low dose of αCGRP induced cAMP synthesis, whereas in non-OA chondrocytes, a high dose of αCGRP was needed to increase the cAMP level, emphasizing the different sensitivity of OA- and healthy chondrocytes to exogenous stimuli. Beside the anti-inflammatory effects of αCGRP and, in part of SP, we suggest that the pro-inflammatory and catabolic effects may be mediated *via* Gq and PLC activation given the observed ERK and AKT pathway activation, which could be blocked by the specific PLC inhibitor U73122. In healthy chondrocytes, ERK and AKT were phosphorylated 5-15 minutes after SP or αCGRP addition. Of note, in OA chondrocytes binding of SP and αCGRP to their receptors only induced phosphorylation of ERK, but not of AKT. Conflicting data concerning AKT signaling in chondrocytes were reported previously. Whereas Appleton et al. postulated articular cartilage degradation after AKT phosphorylation *via* increased MMP-13 expression (48), others reported that AKT signaling promotes matrix synthesis and the survival of chondrocytes, and that a constitutively active AKT signaling pathway in human articular chondrocytes resulted in increased proteoglycan synthesis and Sox9 expression (49, 50). AKT phosphorylation was not changed in OA chondrocytes after SP and αCGRP treatment, again underlining different sensitivity of OA- and non-OA chondrocytes to external stimuli. We therefore suggest that induction in non-OA chondrocytes may depend on the health status or the younger age of the chondrocyte donors. As phosphorylation of ERK was induced in both chondrocyte groups with both neuropeptides, we postulate a common signaling mechanism by SP and αCGRP, which is independent of disease status or donor age. It is well known that MAPK signaling pathways, which include ERK, play an important role in the pathogenesis of OA and that the expression of MMPs in chondrocytes is associated with the activation of MAPK signaling (51, 52).

We want to point out that donors of OA- and healthy chondrocytes were of significant different ages indicating that typical age-related comorbidities are lacking in the donors of the healthy chondrocytes. This shall be kept in mind especially when considering the senescence and apoptosis data which might not only be related to OA but also to the different age of the donors.

## Conclusion

We suggest that there is a pro-inflammatory and destructive effect through active ERK signaling induced by SP and αCGRP in both healthy and OA articular chondrocytes. This catabolic response could be counteracted by an anti-inflammatory and anabolic effect exerted by the cAMP response, particularly as induced by αCGRP addition in non-OA chondrocytes. We suggest that there is a balance between the more catabolic effects of SP and the anabolic effects of αCGRP in healthy joints. However, this balance may be disturbed by the chronic inflammatory milieu in joints of OA patients and shifted toward a catabolic response to SP. Therapeutic approaches that induce a change in one neuropeptide, either SP or CGRP, should therefore include monitoring of the other neuropeptide.

**Figure 8:**
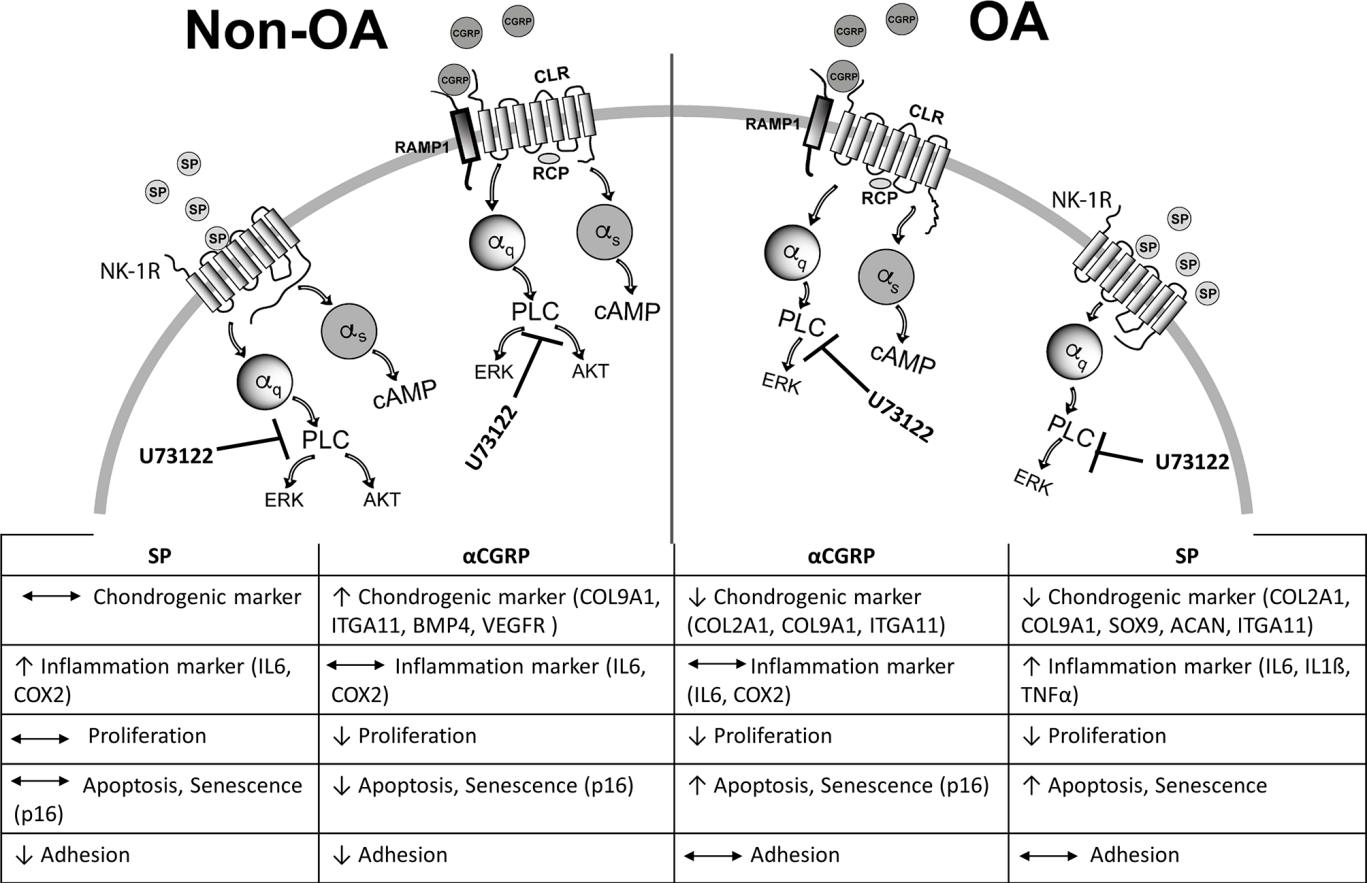
Schematic summary of SP and αCGRP metabolic effects and activated signaling pathways in healthy and OA chondrocytes. Binding of SP and αCGRP to their respective receptors results in different physiological effects in OA- and healthy chondrocytes presumably due to activation of partly different signalling pathways in both chondrocyte groups.

Moreover, we conclude that a change in the involved alpha subunits of the corresponding G-protein-coupled-receptors after binding SP and αCGRP might play a central role in mediating the trophic effects of these neuropeptides on healthy *versus* OA chondrocytes. For healthy chondrocytes, we assume that alpha q, as well as alpha s subunits, can assemble to the receptors for SP or αCGRP thereby affecting downstream signaling pathways, whereas in OA chondrocytes, SP is unable to induce a Gαs assembly to the NK1-R. A schematic overview summarizes signaling events that could occur based on the effects we detected for SP and αCGRP treatment of healthy *versus* OA chondrocytes (**Figure 8**).

## Data Availability Statement

The raw data supporting the conclusions of this article will be made available by the authors, without undue reservation.

## Ethics Statement

The studies involving human participants were reviewed and approved by Ethics committee at the University of Regensburg, Az: 14-101-0189, email: ethikkommission@klinik.ukr.de. The patients/participants provided their written informed consent to participate in this study.

## Author Contributions

SS: Conception and design, analysis and interpretation of the data, drafting of the original article. AE: Seahorse metabolic analysis, review and editing. RB: Graphic support, review and editing. MK: sample acquisition, review and editing. BJ: sample acquisition, review and editing. SG: Conception and design, review and editing, project administration, funding acquisition. All authors contributed to the article and approved the submitted version.

## Funding

This work was funded by a grant from the German Research Foundation (DFG) assigned to SG [GR 1301/18-1].

## Conflict of Interest

The authors declare that the research was conducted in the absence of any commercial or financial relationships that could be construed as a potential conflict of interest.

## Publisher’s Note

All claims expressed in this article are solely those of the authors and do not necessarily represent those of their affiliated organizations, or those of the publisher, the editors and the reviewers. Any product that may be evaluated in this article, or claim that may be made by its manufacturer, is not guaranteed or endorsed by the publisher.
