# Supplementary material for: Substance P and Alpha-Calcitonin Gene-Related Peptide Differentially Affect Human Osteoarthritic and Healthy Chondrocytes
Source: Front Immunol. 2021 Aug 27;12:722884. doi: 10.3389/fimmu.2021.722884 (PMC8430215; doi:10.3389/fimmu.2021.722884)
Supplement: Supplementary file 1 [file DataSheet_1.docx]

**Supplementary figures 1-5 with captions**


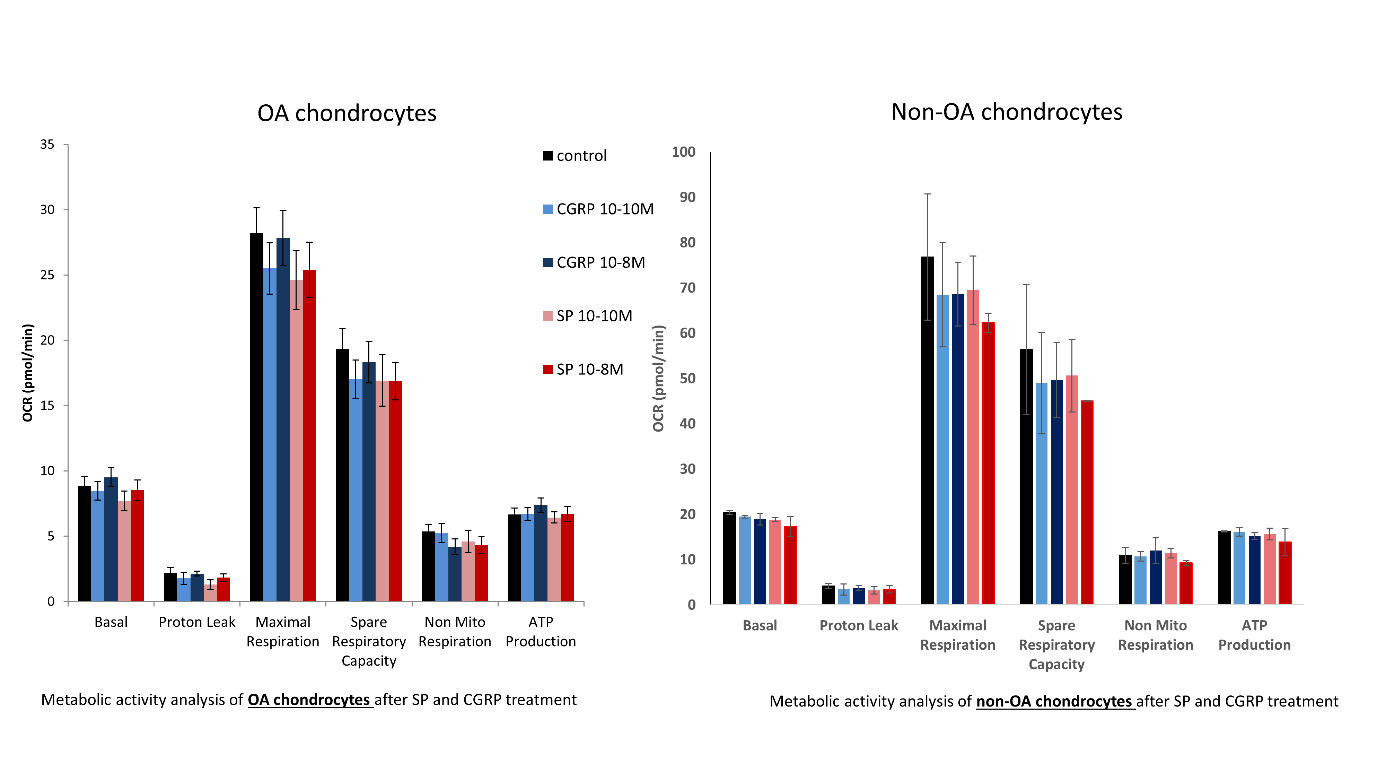
**Supplementary figure 1: Metabolic activity of OA and non-OA chondrocytes**

Metabolic activity was assayed with Seahorse XF Analyzer after SP or αCGRP stimulation in OA and non-OA chondrocytes.

Results are means +/- SD; one sample t-test; n=3


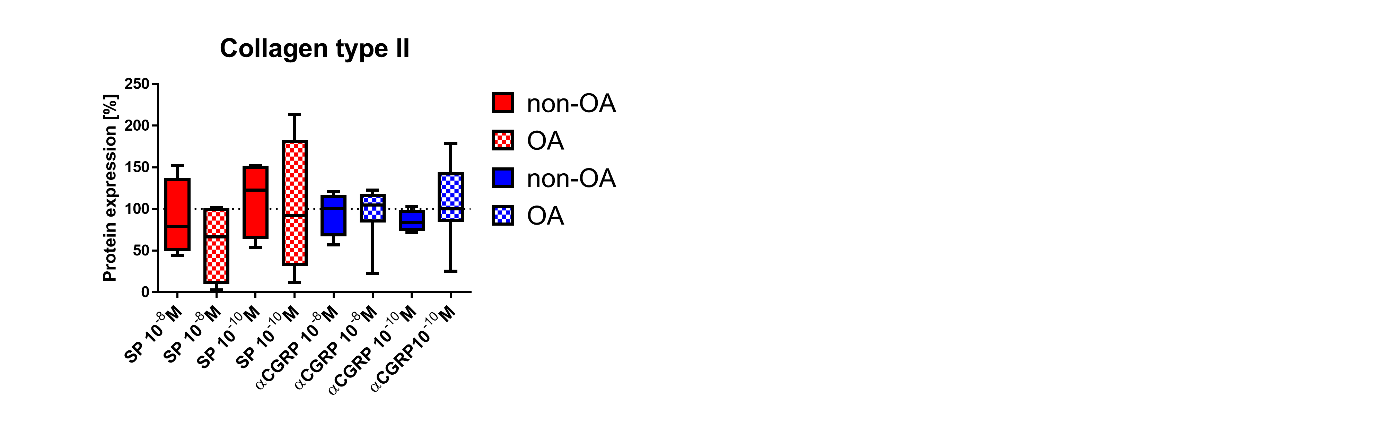


**Supplementary figure 2: Collagen II ELISA**

Collagen type II amount of digested fibrin gels was assayed with an ELISA in OA- and non-OA chondrocytes after stimulation with either 10^-8^ or 10^-10^ M SP or αCGRP.


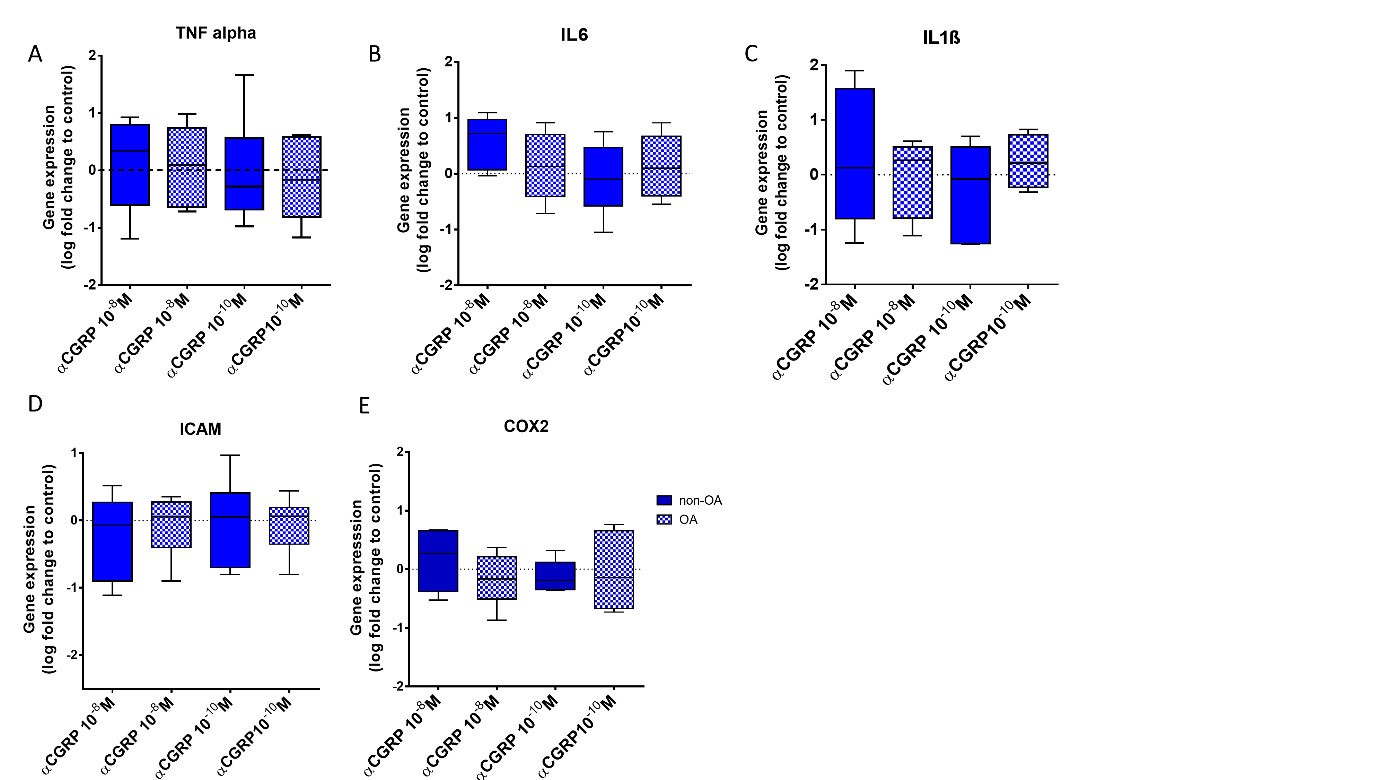


**Supplementary figure 3: Expression of pro-inflammatory genes after αCGRP stimulation of OA- and non-OA chondrocytes**

(A-E) Chondrocytes were treated with either 10^-8^ or 10^-10^M αCGRP for 7 days and then compared to the respective untreated control cells (untreated controls set to 0 or 100% = dotted line). Quantitative RT-PCR analysis shows the gene expression of TNFα, IL6, IL1ß, ICAM and COX2;

Results show median (min to max); one sample t-test; *p≤0,05; n=5-6

**
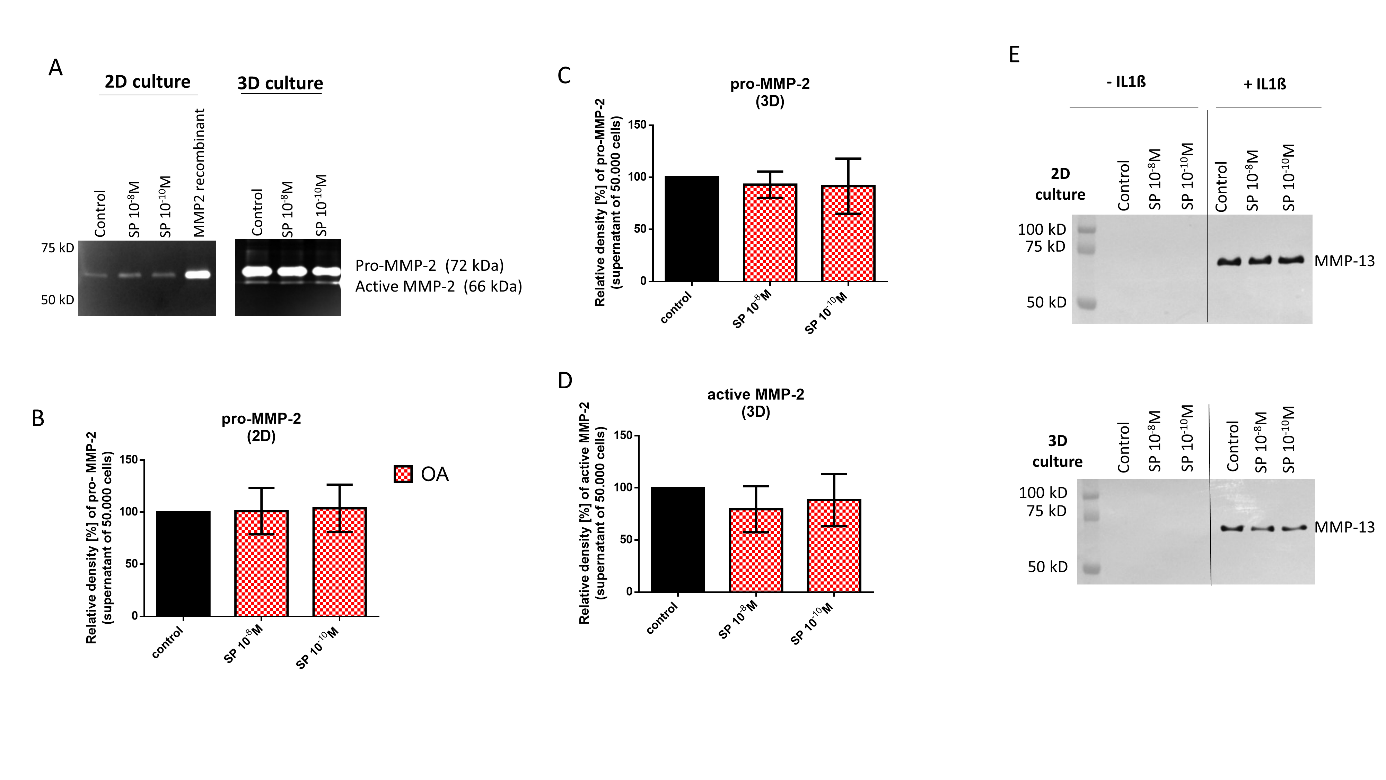
Supplementary figure 4: MMP activity in OA chondrocytes after SP stimulation**

MMP-2 activity in the supernatant of OA chondrocytes, stimulated for 3 (2D) or 7 days (3D fibringels) with 10^-8^ and 10^-10^ M SP, was analysed with gelatin zymography. Representative zymopraphic images for MMP-2 (pro- and active form) is shown (A). Protein expression of pro-MMP-2 in OA chondrocytes cultured in 2D (B), of pro-MMP-2 from OA chondrocytes cultured in 3D (fibrin gels) (C) and of active MMP-2 from OA chondrocytes cultured in 3D (fibrin gels) (D) was determined densitometrically.

n=5-6

(E) Representative Western Blot images of MMP-13. The culture supernatant of OA chondrocytes cultured in 2D and 3D was subjected to SDS-PAGE. Chondrocytes were stimulated with IL1ß as inducer of proteases in addition to 10^-8^ and 10^-10^ M SP stimulation (E). n=5

**
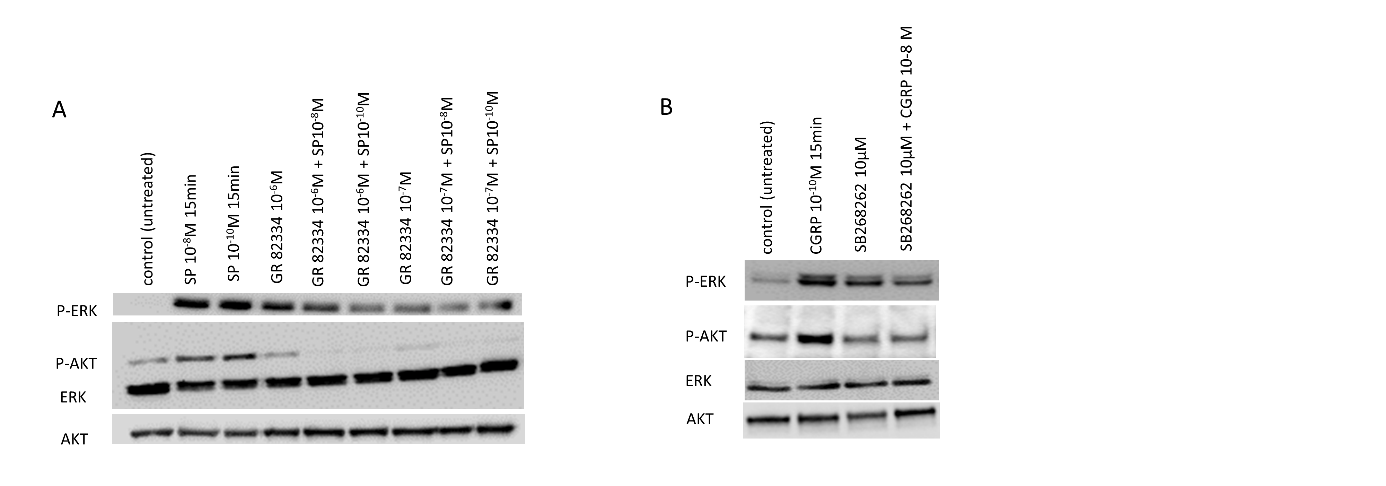
**

**Supplementary figure 5: Inhibition of SP and αCGRP mediated signaling with specific antagonists in C28/I2 cells (human chondrocyte cell line)**

A) SP-specific induction of ERK and AKT was blocked via stimulation with 10^-8^ and 10^-10^ M SP together with NK1 receptor antagonist GR 82,334.

B) αCGRP-specific induction of ERK and AKT was blocked via stimulation with 10^-8^ and 10^-10^ M αCGRP together with non-peptide αCGRP receptor antagonist SB-273779.
